# Zirconium, Hafnium and Tellurium Signatures in Wild Mushrooms from Leicester and Leicestershire, England

**DOI:** 10.3390/jox16040139

**Published:** 2026-07-29

**Authors:** Antonio Peña-Fernández, Tomás Cámara-Pastor, M. Ángeles Peña Fernández, M. Carmen Lobo-Bedmar

**Affiliations:** 1 Area of Legal and Forensic Medicine, Department of Surgery, Medical and Social Sciences, Faculty of Medicine and Health Sciences, University of Alcalá, Ctra. Madrid-Barcelona, Km. 33.600, Alcalá de Henares, 28871 Madrid, Spain; 2 Leicester School of Allied Health Sciences, De Montfort University, Leicester LE1 9BH, UK; 3 Scientific Computation Research Institute (SCRIUR), University of La Rioja, 26006 Logroño, Spain; 4 Department of Biomedical Sciences, Faculty of Pharmacy, University of Alcalá, Ctra. Madrid-Barcelona, Km. 33.6, Alcalá de Henares, 28871 Madrid, Spain; 5 Departamento de Investigación Agroambiental, Madrid Institute for Rural, Agricultural and Food Research and Development (IMIDRA), Finca el Encín, Ctra. Madrid-Barcelona, Km. 38.2, Alcalá de Henares, 28800 Madrid, Spain

**Keywords:** wild mushrooms, zirconium, hafnium, tellurium, technology-associated elements, fruiting bodies, fungal biomonitoring, topsoil, limit of detection, Leicestershire

## Abstract

Zirconium (Zr), hafnium (Hf) and tellurium (Te) are environmentally and technologically relevant elements whose occurrence in wild macrofungi remains insufficiently characterised. This study investigated their concentrations, distribution and multielement relationships in wild mushroom fruiting bodies collected from urban and peri-urban green spaces in Leicester and Leicestershire, England, together with contextual topsoil geochemistry. Acid-recoverable concentrations were determined through inductively coupled plasma mass spectrometry in 121 fruiting-body-level mushroom units and 26 independent site-level composite topsoils. Zr and Hf were detected above their respective working limits of detection in all mushroom units and topsoil composites, whereas Te was detected in 118/121 mushroom units but remained below the working limit of detection in all topsoil composites. Median mushroom concentrations were 170 ng g^−1^ dry weight for Zr, 270 ng g^−1^ for Hf and 110 ng g^−1^ for Te. Comparatively elevated concentrations were observed in *Panaeolina foenisecii* collected from Abbey and Braunstone Parks and in *Mycena citrinomarginata* from Victoria Park, while *Agaricus bitorquis* from St Augustine Road showed lower and less variable concentrations. Park-level concentrations were strongly correlated across the three elements (Spearman’s *ρ* = 0.819–0.885; all BH-adjusted *q* < 0.001), and the first principal component accounted for 93.4% of the total variance, consistent with the strong inter-element correlations. However, contextual soil–mushroom comparisons for Zr and Hf were inconclusive because of limited site overlap and spatial mismatch, and no direct inference regarding environmental source or soil-to-mushroom transfer was supported. These findings establish an exploratory baseline for Zr, Hf and Te in wild mushrooms and demonstrate the value of taxon- and site-resolved fungal biomonitoring for under-characterised environmental elements.

## 1. Introduction

Wild mushrooms have long been used to investigate the occurrence, distribution and biological handling of mineral elements in terrestrial environments. Their fruiting bodies are the visible reproductive structures of mycelial systems that may explore organic matter, mineral soil, litter, woody substrates and microhabitats over variable spatial and temporal scales. Consequently, element concentrations in mushroom fruiting bodies are influenced by fungal taxonomy, trophic strategy, substrate chemistry, soil pH, organic matter, mycelial distribution, tissue structure, element mobility and local geochemical setting [1,2,3,4,5]. Large multielement datasets have also supported the use of macrofungi as bioindicators of environmental quality, while emphasising the need for taxonomic reliability, adequate replication and careful interpretation of species-level patterns [6].

Most research on elemental composition in wild mushrooms has focused on nutritionally relevant elements, potentially toxic metal(loid)s and radionuclides, including Cd, Pb, Hg, As, Cu, Zn, Se and radiocaesium [1,3,7]. By contrast, less frequently monitored lithogenic and technology-associated elements remain comparatively under-characterised in fungal matrices. This is the case for zirconium (Zr), hafnium (Hf) and tellurium (Te), which have been determined in wild mushrooms using different analytical approaches but remain rarely investigated as primary endpoints or as a combined element panel [4,5,8,9,10,11,12,13,14]. As a result, their occurrence, taxon-level variability and relationship with local soil geochemistry remain insufficiently resolved, particularly in urban and peri-urban green spaces.

Zr and Hf are closely related lithophile elements. They share the same common valence state, have similar ionic radii and are commonly associated with resistant accessory minerals, particularly zircon and related heavy mineral phases [15,16,17]. In soils and sediments, their distribution is therefore strongly influenced by parent material, mineral resistance, particle-size effects and detrital mineral inputs. European soil geochemical work has shown that only a small extractable fraction of total Zr and Hf may be released by aqua-regia-type extraction because of the resistant nature of their host minerals [16]. Thus, total or pseudo-total soil concentrations are not necessarily equivalent to the fractions accessible to biological systems.

However, Zr and Hf should not be treated as perfectly invariant in all surface environments. Natural water, colloidal and water–rock interaction studies show that pH, ionic strength, redox conditions, colloids, authigenic mineral surfaces and organic or inorganic complexation can alter their relative behaviour [17,18,19]. The Zr–Hf pair therefore provides a useful geochemical framework for interpreting co-occurrence, while still requiring caution when biological matrices are involved.

Te differs substantially from Zr and Hf in environmental behaviour. It is a rare chalcogen element, usually present at very low concentrations in soils, sediments, natural waters and airborne particles, and its measurement in environmental samples is analytically challenging [20]. Te commonly occurs in environmental systems as tellurite [Te(IV)] and tellurate [Te(VI)], and its geochemistry is often compared with that of Se, although the two elements cannot be assumed to behave identically [20]. Te has also gained attention because of its use in advanced materials, including photovoltaic and thermoelectric technologies and because its environmental occurrence, speciation and biological transformations remain incompletely characterised [20,21]. Soil-to-crop studies indicate that Te can transfer from soils to plant tissues, although transfer varies with crop type and environmental conditions [22]. Comparable evidence for wild mushrooms remains limited.

Zr and Hf are predominantly lithogenic elements whose environmental occurrence is strongly influenced by resistant mineral hosts and surface geochemical processes [15,16,17,18,19], whereas Te is an environmentally scarce chalcogen with recognised ecotoxicological relevance and growing importance in advanced technological applications [20,21]. Considered together, these elements provide an exploratory panel spanning persistent lithogenic constituents and a technology-relevant emerging element. Their occurrence and biological handling in macrofungi remain insufficiently characterised, supporting the relevance of wild mushrooms as exploratory biomonitoring matrices while avoiding assumptions regarding environmental source, exposure pathway or uptake mechanism.

Previous mushroom studies have reported Zr, Hf and Te under markedly different analytical and sampling designs. Activation analysis identified Zr at 5.6 ± 0.7 mg kg^−1^ dw in *Boletus edulis* [8], whereas HNO_3_–HF digestion followed by ICP-MS determined Zr and Hf at 1.1 ± 0.6 and 0.024 ± 0.014 mg kg^−1^ dw, respectively, in caps of *Macrolepiota procera* [9]. In a survey of 20 above-ground and wood-growing species collected near a heavily trafficked road, HNO_3_ digestion and ICP-OES determined Te concentrations of up to 0.30 ± 0.03 mg kg^−1^ dw in *Suillus bovinus*, although Te was evaluated as one endpoint within a 26-element panel [4]. A subsequent ICP-OES study of 21 wood-growing species reported Te concentrations extending from below detection to 11.0 mg kg^−1^ dw, while Hf remained below the applicable detection limit in all species [13]. More recently, ED-XRF analysis of cultivated and wild mushrooms reported Zr concentrations ranging from 14.9 to 168 mg kg^−1^, with the highest concentration in *Lactarius deliciosus* [14].

These studies confirm that Zr, Hf and Te can occur in mushroom fruiting bodies, but direct comparison is limited by differences in taxonomic coverage, tissue pooling, digestion chemistry, analytical platform, detection limits and environmental sampling design. Collectively, they did not provide a focused assessment of the Zr–Hf–Te panel in urban and peri-urban wild mushrooms while simultaneously addressing observations below the detection limit, site-level dependence and the contextual—not microsite-paired—nature of topsoil data. The present study addresses this occurrence and biomonitoring gap without attempting source attribution or inferring species-specific accumulation.

The present study was designed as an exploratory occurrence and biomonitoring investigation of Zr, Hf and Te in wild mushroom fruiting bodies from urban and peri-urban green spaces in Leicester/Leicestershire, England. Mushrooms were treated as the primary biological matrix, whereas topsoils were used as contextual geochemical information rather than as microsite-paired substrates for individual fruiting bodies. The specific objectives were to: (i) quantify Zr, Hf and Te in reconstructed fruiting-body-level mushroom units; (ii) describe detection frequencies and concentration distributions, including observations below the working LoD; (iii) describe concentration patterns among the most represented taxon–site groups, with explicit consideration of their distribution across collection sites; (iv) assess Zr–Hf–Te co-variation using Spearman correlation and principal component analysis (PCA); and (v) compare mushroom data with contextual topsoil patterns while avoiding mechanistic overinterpretation of area-level soil–mushroom associations.

## 2. Materials and Methods

### 2.1. Study Area and Sampling Framework

Wild mushroom fruiting bodies and contextual topsoil samples were collected from public green spaces in Leicester and selected rural or semi-rural sites in Leicestershire, UK. Leicester was considered the main urban study area, while selected Leicestershire sites provided peri-urban or rural green space context. Mushrooms were treated as the primary biological matrix, whereas topsoils were used to describe the broader geochemical setting and were not considered microsite-paired substrates for individual fruiting bodies [2,3,5,23].

The wider environmental monitoring framework included public parks, managed grasslands, open spaces and rural green areas. These sites represent heterogeneous green space environments influenced by urban development, land management, local geology, superficial deposits, historical land use, traffic corridors and natural fruiting availability. Mushroom sampling was therefore opportunistic and field-based: fruiting bodies were collected where they were naturally present during the sampling period. The dataset should not be interpreted as a balanced taxonomic inventory or as a statistically stratified county-wide mushroom survey.

The topsoil sampling framework comprised 850 surface soil field increments collected across 26 sites, including 18 urban parks or open spaces and eight rural Leicestershire locations. Field increments collected within each site were pooled and homogenised to generate one independent site-level composite, yielding 26 environmental sampling units. Two composite-derived analytical or subsampling records were available for each composite, resulting in 52 component records. For Zr and Hf, the two component records were averaged arithmetically to produce one concentration value per site-level composite. Te was below the working LoD in both component records for every composite. The 52 component records were retained only for analytical traceability and QA/QC and were not treated as independent environmental observations. The spatial relationship between the mushroom collection sites and the contextual topsoil composite sites is shown in Supplementary Figure S1.

### 2.2. Wild Mushroom Collection, Labelling and Taxonomic Identification

Wild mushroom fruiting bodies were collected between September and November 2019 during dry weather, with ambient temperatures of approximately 8–17 °C. Fruiting bodies were visually located across each park or green space area and collected according to natural availability. The geographical position of each collection point was recorded using a handheld Global Positioning System device. The opportunistic sampling protocol did not impose a predefined minimum separation between fruiting bodies. Although each collection point was georeferenced, the spatial extent and genotypic identity of the underlying mycelial systems were not determined. Consequently, multiple fruiting bodies collected within the same park may represent spatially clustered observations and cannot necessarily be regarded as independent fungal genets.

Specimens were handled with nitrile gloves to minimise external contamination and to avoid direct contact with potentially toxic or inedible taxa. Where possible, complete fruiting bodies were collected by carefully removing the stem from the upper soil or substrate. Each specimen was placed in an individually labelled sealed polypropylene bag, transported to the laboratory and processed separately.

Taxonomic identification was based initially on macroscopic and microscopic characteristics and, where suitable material was available, supported by DNA barcoding. Genomic DNA was extracted from approximately 100 mg of frozen homogenised mushroom material using the DNeasy Plant Mini Kit^®^ (Qiagen Inc., Germantown, MD, USA), following the manufacturer’s protocol. The internal transcribed spacer region of fungal ribosomal DNA was amplified using ITS1/ITS4 primers, sequenced and compared with reference sequences for species confirmation, consistent with the use of ITS as the standard DNA barcode region for fungi [24]. For some specimens, DNA extraction or repeated ITS amplification was unsuccessful, most likely because of degraded DNA and/or PCR inhibition. These records were retained as unknown or unidentified analytical units for overall descriptive analyses but were not used for taxon-specific biological interpretation.

The most represented taxa in the final reconstructed dataset were *Panaeolina foenisecii*, *Mycena citrinomarginata*, *Agaricus bitorquis*, *Coprinopsis atramentaria* and an unresolved/unknown analytical subset. *Panaeolina foenisecii* was interpreted as a saprotrophic grassland species frequently associated with nutrient-rich lawns, meadows, parks, gardens, pastures and roadsides, where it contributes to decomposition of organic matter and nutrient cycling [25]. This ecological profile is relevant to the managed green space habitats represented in the present study.

### 2.3. Mushroom Cleaning, Tissue Separation and Homogenisation

In the laboratory, visible debris, insects and adherent soil particles were removed manually from each fruiting body. Samples were washed with tap water, rinsed with deionised water and finally rinsed with Milli-Q ultrapure water produced using a Milli-Q^®^ Direct 8 system (Merck Millipore, Darmstadt, Germany; resistivity 18.2 MΩ·cm). Comparable cleaning, drying and homogenisation procedures are commonly used in multielement studies of wild and cultivated mushrooms to minimise superficial contamination and obtain representative analytical material [4,9,10,11,14].

Where sufficient biomass was available, fruiting bodies were separated into cap and stem fractions before drying, allowing tissue-specific information to be retained. When biomass was limited, whole fruiting bodies were processed intact to prioritise taxon- and site-level analytical coverage. Tissue separation is useful because element partitioning between cap and stem can vary among elements and taxa [3,5,7,11].

Cleaned mushroom material was oven-dried at 50 °C. The dried material was cryogenically homogenised with liquid nitrogen using a ceramic mortar and pestle to obtain a fine, visually uniform powder. No sieving step was performed. The homogenised material was thoroughly mixed and stored at −80 °C in capped polypropylene tubes until digestion.

Reusable equipment, including forceps, spatulas, mortars, pestles and digestion vessels, was cleaned between samples using laboratory detergent, deionised water, 5% *v*/*v* nitric acid and repeated rinsing with Milli-Q ultrapure water. Glassware was avoided where possible for trace-element work.

### 2.4. Fruiting-Body-Level Reconstruction

The original analytical dataset contained records from directly analysed whole fruiting bodies and from cap and stipe fractions analysed separately. For the main analysis, a reconstructed fruiting-body-level unit was defined as one analytical record representing one fruiting body: either the concentration measured directly in a whole fruiting body or the unweighted arithmetic mean of the matched cap and stipe concentrations from the same fruiting body. The reconstruction was therefore performed at the dataset level to prevent paired tissues from the same fruiting body from being treated as independent observations. The cap–stipe averages were not tissue mass-weighted because complete cap and stipe dry masses were not available for all paired records.

Single-tissue records without a corresponding paired tissue were excluded from the main fruiting-body-level dataset. The final main dataset comprised 121 reconstructed fruiting-body-level units. This dataset was used for detection summaries, descriptive statistics, descriptive taxon–site summaries, Spearman correlation analysis and principal component analysis.

This reconstruction was used to avoid treating paired cap and stipe measurements from the same fruiting body as independent observations. Because tissue masses were unavailable, paired concentrations were combined using an unweighted arithmetic mean; the resulting values therefore represent reconstructed fruiting-body-level analytical units rather than mass-weighted whole-fruiting-body concentrations. Sensitivity analyses based on directly analysed whole fruiting bodies and paired tissues, including paired cap–stipe comparisons, are reported in Supplementary Table S1.

### 2.5. Mushroom Mineralisation and ICP-MS Determination

Dried and homogenised mushroom powders were mineralised using a closed-vessel acid digestion procedure in pre-cleaned Teflon digestion vessels. Microwave-assisted digestion was not used for the mushroom matrix. Where sufficient material was available, approximately 0.500 g of dry mushroom powder was accurately weighed into each vessel. Five millilitres of 65% (*v*/*v*) nitric acid were added to each sample, followed by 1 mL of 30% (*v*/*v*) hydrogen peroxide, both Suprapur^®^ grade (Merck KGaA, Darmstadt, Germany). The sealed vessels were maintained at room temperature for 8 h and subsequently heated in a laboratory oven at 96 °C for 12 h. Nitric acid–hydrogen peroxide digestion is widely used for trace-element determination in biological and fungal matrices because it supports efficient oxidation of organic material prior to instrumental analysis [4,9,10,11].

After digestion and cooling, digestates were filtered through Whatman™ Grade 42 ashless quantitative filter paper (Cytiva, Marlborough, MA, USA) and diluted to a final volume of 25 mL with Milli-Q ultrapure water produced using a Milli-Q^®^ Direct 8 purification system (Merck Millipore, Darmstadt, Germany). Before ICP-MS analysis, an aliquot of each digest was passed through a 0.45 µm PTFE syringe filter to remove any residual fine particulate material and protect the sample introduction system. Accordingly, the reported mushroom concentrations represent acid-recoverable concentrations in the filtered digestates. They should not be interpreted as complete total concentrations or as unequivocally intracellular concentrations, because complete dissolution of refractory mineral particles and complete removal of microscopically fine surface-associated material were not independently demonstrated.

Zr, Hf and Te were quantified through inductively coupled plasma mass spectrometry using a PerkinElmer NexION 2000B ICP-MS system (PerkinElmer Inc., Waltham, MA, USA) coupled with an Elemental Scientific prepFAST M5 autosampler (Elemental Scientific Inc., Omaha, NE, USA). Instrument control and data acquisition were performed using Syngistix™ Software for ICP-MS, version 3.2 (PerkinElmer Inc., Waltham, MA, USA). ICP-MS provides the sensitivity required for low-level multielement determination in biological and environmental matrices, including less frequently monitored trace and ultratrace elements [4,10,20]. Mushroom concentrations were converted to dry weight concentrations and reported as ng g^−1^ dw.

### 2.6. Contextual Topsoil Analysis

The topsoil dataset was used to support interpretation of the broader geochemical setting of the monitored parks and green spaces. It was not used to infer direct individual soil-to-mushroom uptake. The full topsoil framework was based on surface topsoil collected from the upper 0–5 cm horizon. Soil material was air-dried, sieved through a 2 mm stainless steel mesh, pooled into site-level composites and homogenised.

Topsoil aliquots were subjected to microwave-assisted digestion with reverse aqua regia, comprising 6 mL of 69% nitric acid and 2 mL of 37% hydrochloric acid (3:1 *v*/*v* HNO_3_:HCl)—both Suprapur^®^ grade (Merck, Darmstadt, Germany)—using a Multiwave GO microwave digestion system (Anton Paar GmbH, Graz, Austria). The digestion programme consisted of an initial temperature ramp at 10 °C min^−1^ to 150 °C, followed by a 2 min holding period, and a second ramp at 3.3 °C min^−1^ to 190 °C, followed by a 45 min holding period. After cooling, the digestates were filtered through Whatman™ Grade 42 ashless quantitative filter paper and diluted to a final volume of 50 mL with Milli-Q ultrapure water. Before ICP-MS determination, the mineralised samples were diluted in 5% *v*/*v* nitric acid and filtered through 0.45 µm syringe filters.

The reverse aqua regia digestion was interpreted as a pseudo-total extraction of the acid-extractable or recoverable soil element pool, not as complete silicate dissolution. This distinction is particularly relevant for Zr and Hf because these elements are commonly hosted by resistant accessory mineral phases, especially zircon and related heavy minerals, and may be only partly released by this acid digestion [15,16,17].

### 2.7. Soil Physicochemical Characterisation

Soil physicochemical properties and texture were determined in homogenised composite topsoil material at IMIDRA using Spanish official soil analytical protocols [26]. The measured parameters were pH, electrical conductivity, moisture content, organic matter and soil texture. These variables were used as contextual edaphic descriptors because pH, organic matter, moisture and texture can influence element sorption, desorption, solubility, fine particle retention and apparent soil-to-biota transfer [3,5,27,28].

After air-drying and sieving through a 2 mm mesh, pH and electrical conductivity were measured in soil–water suspensions using a 1:2.5 *w*/*v* ratio of topsoil to deionised water. Briefly, 10 g of homogenised topsoil was mixed with 25 mL of deionised water and agitated for 20 min before measurement with calibrated probes. Moisture content was determined gravimetrically after drying at 105 °C to constant weight. Organic matter was determined using the Walkley–Black dichromate oxidation method. Soil texture was determined on the homogenised <2 mm fraction using the Bouyoucos hydrometer method, allowing estimation of sand, silt and clay fractions.

### 2.8. Analytical Calibration, Verification and Quality Assurance

Analytical calibration was performed using commercial multielement standards supplied by CPAchem Ltd. (Bogomilovo, Stara Zagora, Bulgaria), whereas an independent commercial aqueous quality control solution supplied by QMx Laboratories Ltd. (Thaxted, Essex, UK) was used to verify instrumental performance. Before ICP-MS measurement, mushroom and topsoil digestates were manually diluted 11-fold with Milli-Q ultrapure water. Calibration standards, quality control solutions and samples were analysed under the same instrumental conditions using a PerkinElmer NexION 2000B ICP-MS system (PerkinElmer Inc., Waltham, MA, USA) coupled with an Elemental Scientific prepFAST M5 autosampler (Elemental Scientific Inc., Omaha, NE, USA). Instrument control and data acquisition were performed using Syngistix™ Software for ICP-MS, version 3.2.

Zirconium was monitored as ^90^Zr in helium kinetic energy discrimination mode, using a helium flow rate of 3.9 mL min^−1^. Hafnium and Te were monitored as ^178^Hf and ^130^Te, respectively, in standard mode. Measurements were acquired using peak hopping scanning, with 20 sweeps per reading, one reading per replicate and five instrumental replicates per analysed solution. Dwell and integration times were 20 ms and 400 ms for ^90^Zr, 15 ms and 300 ms for ^178^Hf, and 25 ms and 500 ms for ^130^Te, respectively. A software-defined interference correction incorporating the ^129^Xe and ^137^Ba signals was applied to ^130^Te:

^130^Te _corrected_ = ^130^Te _measured_ − 0.154201 × ^129^Xe − 0.009420 × ^137^Ba.

Indium-115 (^115^In) was continuously introduced by the prepFAST system as an internal standard. Separate ^115^In signals were assigned to analytes measured in helium KED and standard modes. Internal standard variation was generally below 10% across the samples and was used to monitor instrumental stability and matrix-related changes in signal response.

For the mushroom analytical batch, 10-point linear calibration curves extended to an upper calibration standard of 77.759 µg L^−1^ for Zr, Hf and Te and showed correlation coefficients of at least 0.999. Instrumental detection limits were 0.007 µg L^−1^ for Zr, 0.001 µg L^−1^ for Hf and 0.014 µg L^−1^ for Te. The corresponding working dry weight LoDs, calculated by converting the instrumental limits according to sample mass, final digest volume and dilution factor, were 0.294 ng g^−1^ dw for Zr, 0.823 ng g^−1^ dw for Hf and 5.269 ng g^−1^ dw for Te. The results were classified relative to the matrix-specific working LoD. A separate limit of quantification was not established; therefore, “detected” denotes a measured result above the applicable working LoD and does not imply verification against an independently established limit of quantification.

Instrumental performance for the mushroom batch was evaluated using the independent QCB 280 aqueous quality control solution. The solution was automatically diluted twofold using the prepFAST system before measurement, corresponding to a nominal concentration of approximately 140 µg L^−1^ introduced for analysis. This concentration remained above the upper calibration standard of 77.759 µg L^−1^; consequently, the QC concentrations were estimated by extrapolation of the linear calibration models. The 10-point calibration curves showed coefficients of determination of at least 0.999, with no evidence of detector saturation or visible departure from linearity in the archived instrumental records. After accounting for the automatic dilution, mean back-calculated QC concentrations were 287 ± 9.39 µg L^−1^ for Zr, 286 ± 17.5 µg L^−1^ for Hf and 302 ± 20.3 µg L^−1^ for Te. These values corresponded to approximate instrumental recoveries of 102%, 102% and 108%, with relative standard deviations of 3.27%, 6.11% and 6.73%, respectively.

For the topsoil analytical batch, 10-point linear calibration curves extended to a nominal upper calibration standard of 117 µg L^−1^ for Zr, Hf and Te and showed coefficients of determination of at least 0.999. Instrumental detection limits were 0.005 µg L^−1^ for Zr, 0.002 µg L^−1^ for Hf and 0.014 µg L^−1^ for Te. The corresponding working dry weight LoDs were 0.0131 mg kg^−1^ dw for Zr, 0.0278 mg kg^−1^ dw for Hf and 0.0556 mg kg^−1^ dw for Te.

Instrumental performance for the topsoil batch was evaluated using the independent QCB 274 aqueous quality control solution. Following automatic twofold dilution, the nominal concentration introduced for analysis was approximately 137 µg L^−1^, approximately 17% above the nominal upper calibration standard of 117 µg L^−1^. The QC concentrations were therefore estimated through modest extrapolation of the linear calibration models. The calibration records showed no evidence of detector saturation or visible departure from linearity. Mean back-calculated concentrations were 264 ± 4.10 µg L^−1^ for Zr, 270 ± 1.50 µg L^−1^ for Hf and 276 ± 15.7 µg L^−1^ for Te. These corresponded to approximate instrumental recoveries of 96.4%, 98.6% and 101%, with relative standard deviations of 1.55%, 0.56% and 5.68%, respectively.

Each analytical sequence included initial 2% *v*/*v* HNO_3_ wash solutions, calibration standards, an independent quality control solution and additional acid washes. Wash solutions were analysed after approximately every 20 samples, and the quality control solution was repeated later in the sequence. The prepFAST injection loop was rinsed with clean 2% *v*/*v* HNO_3_ between measurements to minimise potential carry-over. Review of the analytical sequences did not indicate appreciable carry-over. As an additional quality assurance step, analyte assignments, dilution factors and dry weight conversion calculations were verified before statistical analysis.

The 2% *v*/*v* HNO_3_ wash solutions were used as instrumental blanks and to monitor potential carry-over. Four initial wash measurements in the mushroom analytical sequence gave mean concentrations of 0.03751 ± 0.00018 µg L^−1^ for Zr, 0.17431 ± 0.00049 µg L^−1^ for Hf and 0.10208 ± 0.00315 µg L^−1^ for Te. No blank subtraction was applied to the sample results. The instrumental detection limits used in this study were those reported in the archived Syngistix calibration outputs: 0.007 µg L^−1^ for Zr, 0.001 µg L^−1^ for Hf and 0.014 µg L^−1^ for Te. Values below the corresponding working dry weight LoDs were treated as observations below the working LoD. Te was below the working LoD in all contextual topsoil records and was therefore reported as not detected above the applicable working LoD under the analytical conditions used. This result was interpreted cautiously, considering the low environmental abundance of Te and the analytical challenges associated with its determination in soils, sediments and other solid environmental matrices [20].

The independent aqueous quality control solutions were used to verify instrumental calibration consistency and repeatability. Because they were not subjected to the mushroom or topsoil digestion procedures, they did not assess digestion efficiency or matrix-specific recovery. Matrix-matched procedural blanks, certified reference materials, matrix spike recoveries and independent replicate digestions were not available for these analytical batches. Full calibration ranges, instrumental detection limits, working dry weight LoDs and instrumental QC summaries are reported in Supplementary Table S2.

### 2.9. Treatment of Values Below the Working LoD and Descriptive Statistics

Values below the applicable working limit of detection (LoD) were treated statistically as left-censored observations. Detection frequency and the proportion of values below the working LoD were evaluated separately for mushrooms and topsoils. Zr and Hf were detected in all 121 reconstructed fruiting-body-level units and in all 26 independent site-level topsoil composites. Te was detected in 118/121 mushroom units. In the topsoil dataset, Te was below the working LoD in all 52 component analytical records and was therefore not detected above the working LoD in any of the 26 site-level composites.

Formal descriptive summaries for Te were obtained using a lognormal regression-on-order-statistics (ROS) framework. Values below the working LoD were retained as left-censored observations for detection-frequency and range reporting. For analyses requiring complete numerical input, the three Te observations below the working LoD were estimated using a bound-constrained ROS procedure in which their ordered probabilities were placed within the fitted lognormal distribution conditional on Te being below the working LoD. This ensured that all three estimates remained below 5.269 ng g^−1^ dw. The constrained estimates were used to calculate the arithmetic and geometric means and to provide complete numerical input for PCA.

The empirical 95th and 97.5th percentiles were calculated as sample quantiles on the original concentration scale and were not derived from a fitted lognormal distribution. The lognormal assumption was used only within the constrained ROS procedure applied to the three left-censored Te observations. Because these observations were confined to the lower tail of the distribution, the median, interquartile range, and empirical 95th and 97.5th percentiles were invariant to their exact estimated values. The arithmetic means for Zr and Hf were calculated directly from the observed concentrations, whereas the arithmetic mean for Te was calculated directly from the ROS-completed concentrations on the original scale. No arithmetic mean was obtained through back-transforming a mean calculated on the log scale; therefore, no back transformation bias correction was required.

For the site-level Spearman analyses, each of the three Te observations below the working LoD occurred below the detected concentrations defining the median of its corresponding park. Consequently, the park-level Te medians and their rank order were unaffected by the unknown concentrations below the working LoD. Soil Te was excluded from quantitative distributional summaries, soil–mushroom ratios, correlation analysis and ordination because it remained below the working LoD in all 52 component analytical records and all 26 independent composites. Elements for which all measurements were below the working LoD were not assigned quantitative descriptive, correlation, ordination or ratio estimates [29,30].

For each element with observed or statistically estimable concentrations, descriptive statistics included N, the number and percentage of detected observations, the percentage below the working LoD, LoD, range, detected range where relevant, arithmetic mean, geometric mean, median, interquartile range, 95th percentile and 97.5th percentile. For elements for which all measurements were below the working LoD, only detection frequency, percentage below the working LoD and the matrix-specific LoD were reported. Because environmental and biological concentration data are typically right-skewed, medians, interquartile ranges and percentile-based summaries were emphasised over arithmetic means.

Descriptive concentration summaries were prepared for taxa or analytical groups represented by at least five reconstructed fruiting-body-level units. A complete taxon-by-site cross-tabulation was used to assess the distribution of each group across the collection sites. Because most principal taxa were represented at a single site, site and taxonomy could not be estimated as independent explanatory factors, and formal site-adjusted comparisons among taxa were therefore not performed. The observed concentration distributions were interpreted as descriptive taxon–site patterns. *Panaeolina foenisecii* was the only principal taxon collected at more than one site, and its site-specific median concentrations were examined descriptively.

Paired cap–stipe concentrations were compared using two-sided Wilcoxon signed-rank tests. *p* values for the three elements were adjusted using the Benjamini–Hochberg procedure, and effect sizes were expressed as rank biserial correlations. Bias-corrected and accelerated 95% bootstrap confidence intervals were calculated using 10,000 resamples, with the park, independent site-level composite or paired fruiting body used as the resampling unit, as appropriate.

### 2.10. Multivariate Analysis and Soil–Mushroom Contextual Comparisons

To address potential dependence among fruiting bodies collected within the same location, the primary Spearman rank correlations among Zr, Hf and Te were calculated using site-level median concentrations as independent ecological units (*N* = 13 parks), rather than treating all 121 reconstructed fruiting-body-level units as spatially independent observations. Spearman correlation was selected because the site-level concentrations were non-normally distributed and the analysis was intended to evaluate monotonic co-variation. Each of the three Te observations below the working LoD occurred below the detected concentrations defining the median of its corresponding park; consequently, the site-level Te medians and their rank order were invariant to the unknown concentration below the LoD. These analyses were interpreted as exploratory site-level associations because the parks differed in taxonomic composition and number of collected fruiting bodies.

Principal component analysis (PCA) was performed on log_10_-transformed and standardised Zr, Hf and Te concentrations in the reconstructed fruiting-body-level dataset (*N* = 121). Observed Zr and Hf concentrations were used directly, whereas the three Te observations below the working LoD were completed using the bound-constrained ROS procedure described in Section 2.9. Log transformation was used to reduce the influence of right-skewed distributions, and standardisation ensured that the ordination reflected correlation structure rather than absolute concentration scale. PCA has been used in multielement mushroom studies to explore co-variation among elements and samples [4,9,11,12]. In the present study, it was used to provide a joint sample-level visualisation of Zr–Hf–Te co-variation and dispersion, complementing the site-level Spearman correlations. Because only three strongly correlated variables were included, the ordination was not interpreted as independent evidence of a common environmental source, uptake mechanism or biological regulation.

Exploratory same-element comparisons between mushroom and contextual topsoil concentrations were performed for Zr and Hf at the overlapping collection sites. Mean-based comparisons included six parks, whereas median-based sensitivity comparisons included seven parks. Te was excluded because it was below the working LoD in all contextual topsoil composites. Because the topsoil composites were not microsite- or rhizosphere-paired with individual mushroom fruiting bodies, and because the numbers of overlapping sites were limited, these analyses were treated strictly as descriptive contextual comparisons rather than as tests of soil-to-mushroom transfer or uptake.

Apparent mushroom/contextual topsoil concentration ratios for Zr and Hf were calculated after unit harmonisation using the independent contextual topsoil composite concentration available for each park and the corresponding park-level median mushroom concentration. These ratios are reported in Supplementary Table S3 solely as descriptive area-level contextual indicators. They were not interpreted as bioconcentration factors, transfer factors, bioaccumulation factors or mechanistic uptake coefficients because the contextual topsoil composites were not paired with the microsubstrates accessed by the fungal mycelia [2,3,5].

Where national geochemical context was considered for Zr and Hf, values from the *Advanced Soil Geochemical Atlas of England and Wales* were used only as external reference information [15]. This comparison was treated cautiously because the Atlas is based on national-scale topsoil geochemical data generated using total X-ray fluorescence methods, whereas the present topsoil results represent acid-recoverable concentrations obtained using reverse aqua regia digestion. Therefore, any comparison with British Geological Survey data should be interpreted as contextual geochemical framing, not as a direct method-equivalent enrichment assessment or evidence of mushroom uptake.

Data processing and statistical analyses were performed in R statistical software (version 4.3.1). Figures were generated from the reconstructed fruiting-body-level dataset and the contextual topsoil dataset.

## 3. Results

### 3.1. Detection Frequency and Overall Mushroom Concentrations

Zr and Hf were detected in all 121 reconstructed fruiting-body-level mushroom units. Te was detected in 118/121 units, corresponding to a detection frequency of 97.5%. Thus, Zr and Hf were detected above their respective working LoDs in all reconstructed mushroom units, while Te was detected above its working LoD in 118/121 units, with only three observations below the working LoD (Table 1).

Zr concentrations ranged from 28 to 3200 ng g^−1^ dw, with a median of 170 ng g^−1^ dw, a mean of 320 ng g^−1^ dw and a geometric mean of 170 ng g^−1^ dw. The interquartile range was 61–380 ng g^−1^ dw. The 95th and 97.5th percentiles were 1100 and 1500 ng g^−1^ dw, respectively.

Hf concentrations ranged from 53 to 6000 ng g^−1^ dw, with a median of 270 ng g^−1^ dw, a mean of 680 ng g^−1^ dw and a geometric mean of 330 ng g^−1^ dw. The interquartile range was 99–850 ng g^−1^ dw. The 95th and 97.5th percentiles were 2800 and 3700 ng g^−1^ dw, respectively.

Using the complete working dataset required for exploratory analyses, the Te working concentration range extended from <5.269 to 3300 ng g^−1^ dw, with a detected minimum of 6.8 ng g^−1^ dw. The median concentration was 110 ng g^−1^ dw and the interquartile range was 46–420 ng g^−1^ dw. Using the constrained-ROS-completed dataset, the arithmetic mean concentration was 350 ng g^−1^ dw and the geometric mean was 140 ng g^−1^ dw. The 95th and 97.5th percentiles were 1700 and 2100 ng g^−1^ dw, respectively. The detected-only sensitivity median was similar, at 120 ng g^−1^ dw, supporting the limited influence of the three observations below the working LoD.

All three elements showed right-skewed distributions, with upper tail enrichment in a subset of fruiting bodies. This was particularly evident for Hf and Te, although Zr also showed a wide concentration range.

The sensitivity analyses did not identify significant cap–stipe differences for Zr or Hf after correction for multiple comparisons. Te showed a moderate tissue-related difference, but this result was considered exploratory because the paired subset was dominated by a single taxon–site group (Supplementary Table S1).

### 3.2. Concentration Patterns Among Principal Taxon–Site Groups

Descriptive concentration profiles varied among the principal taxon–site groups represented in the dataset (Table 2 and Supplementary Table S4). *Panaeolina foenisecii* was collected at Abbey Park (*n* = 23) and Braunstone Park (*n* = 5), whereas *Mycena citrinomarginata*, *Coprinopsis atramentaria* and *Agaricus bitorquis* were collected at Victoria Park (*n* = 22), Bradgate Park (*n* = 8) and St Augustine Road (*n* = 22), respectively. The unknown/unidentified group was collected at Castle Garden (*n* = 17). Because most principal taxa were represented at a single collection site, the observed concentration profiles are interpreted as descriptive taxon–site patterns rather than as independent taxonomic effects.

Across the complete *P. foenisecii* group (*n* = 28; Abbey Park and Braunstone Park), median concentrations were 390 ng g^−1^ dw for Zr, 950 ng g^−1^ dw for Hf and 540 ng g^−1^ dw for Te. Maximum concentrations reached 3200 ng g^−1^ dw for Zr, 4900 ng g^−1^ dw for Hf and 2300 ng g^−1^ dw for Te.

For *M. citrinomarginata* (*n* = 22; Victoria Park), median concentrations were 390 ng g^−1^ dw for Zr, 730 ng g^−1^ dw for Hf and 360 ng g^−1^ dw for Te. Maximum concentrations reached 2000 ng g^−1^ dw for Zr, 6000 ng g^−1^ dw for Hf and 3300 ng g^−1^ dw for Te.

For *C. atramentaria* (*n* = 8; Bradgate Park), median concentrations were 260 ng g^−1^ dw for Zr, 520 ng g^−1^ dw for Hf and 100 ng g^−1^ dw for Te. The interquartile ranges were comparatively wide, particularly for Hf and Te, within this sampled taxon–site group.

For *A. bitorquis* (*n* = 22; St. Augustine Road), median concentrations were 56 ng g^−1^ dw for Zr, 98 ng g^−1^ dw for Hf and 44 ng g^−1^ dw for Te. The concentration distributions were comparatively narrow within this sampled taxon–site group.

The unknown/unidentified group (*n* = 17; Castle Garden) showed median concentrations of 89 ng g^−1^ dw for Zr, 110 ng g^−1^ dw for Hf and 54 ng g^−1^ dw for Te. This group was retained in the overall descriptive analyses but was not used for taxonomic interpretation.

Because *P. foenisecii* was the only principal taxon represented at more than one collection site, its site-specific medians were also examined descriptively. Median concentrations at Abbey Park and Braunstone Park were 403 and 357 ng g^−1^ dw for Zr, 966 and 926 ng g^−1^ dw for Hf, and 539 and 535 ng g^−1^ dw for Te, respectively. This limited comparison is not interpreted as evidence of a general species-level concentration profile.

### 3.3. Site-Level Co-Variation in Zr, Hf and Te in Mushrooms

Using site-level median concentrations as independent ecological units (*N* = 13), Zr, Hf and Te showed strong positive associations. Spearman’s *ρ* was 0.863 (95% BCa CI: 0.381–0.983) for Zr–Hf, 0.819 (95% BCa CI: 0.269–0.961) for Zr–Te and 0.885 (95% BCa CI: 0.419–0.994) for Hf–Te; all three associations had *p* < 0.001 and BH-adjusted *q* < 0.001 (Supplementary Table S5). The three Te observations below the working LoD did not affect the site-level medians or their rank order because each occurred below the detected concentrations defining the corresponding park median.

These results describe co-variation among site-level mushroom concentration profiles. They should be considered exploratory because taxonomic composition and sample numbers differed among parks, and they do not establish a common environmental source, direct uptake pathway or shared biological regulation.

### 3.4. Principal Component Analysis

**Figure 1:**
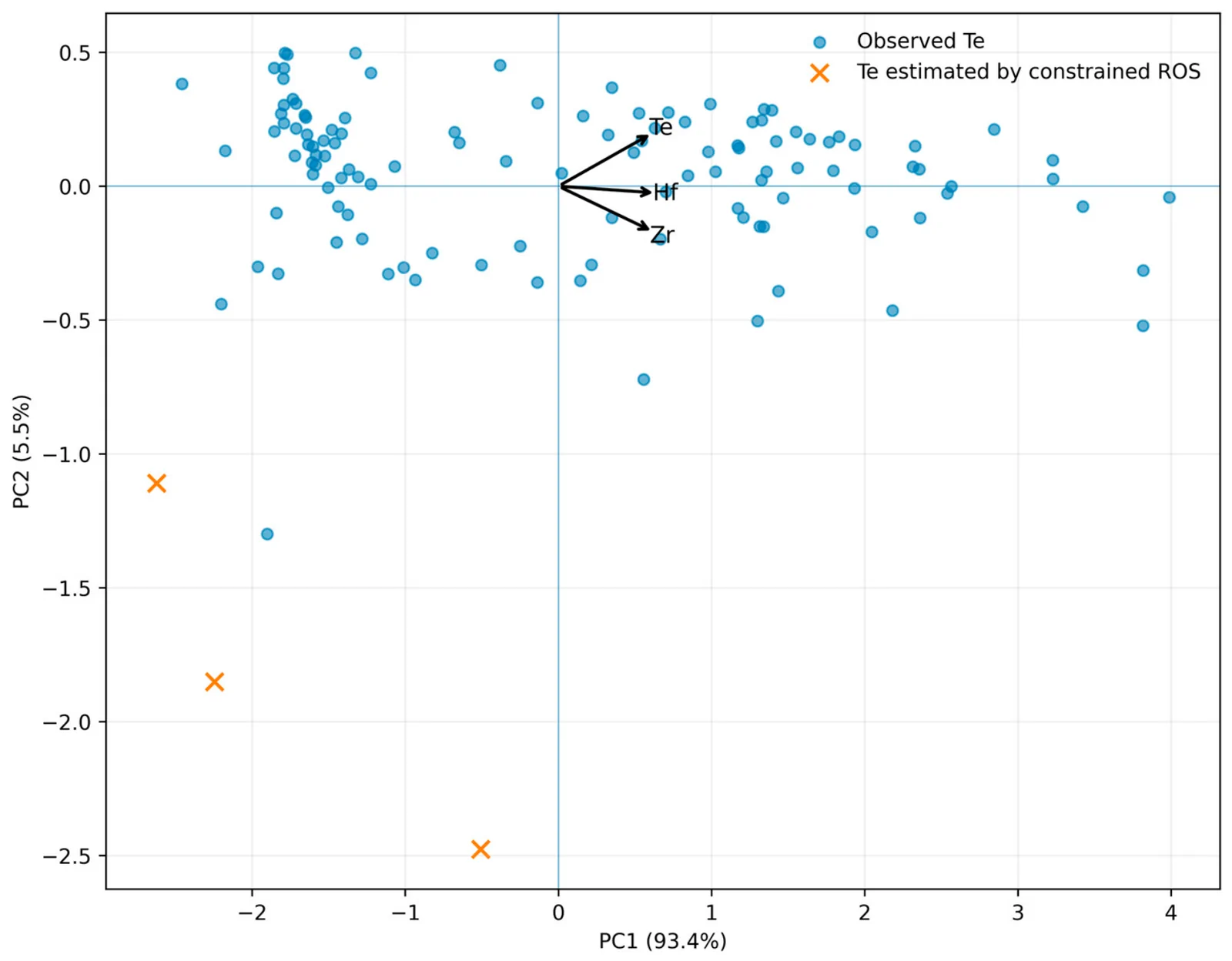
Exploratory principal component analysis biplot for log_10_-transformed and standardised Zr, Hf and Te concentrations in reconstructed mushroom fruiting-body-level units (*N* = 121). Observed Zr and Hf concentrations were used directly, and the three Te observations below the working LoD were estimated using bound-constrained regression on order statistics before transformation and standardisation. PC1 explained 93.4% of the total variance and PC2 explained 5.5%. Circles represent units with observed Te concentrations, whereas crosses represent units with Te estimated using constrained ROS. The ordination provides a joint visualisation of three-element co-variation and should not be interpreted as evidence of a common environmental source, uptake mechanism or biological regulation.

The exploratory PCA provided a joint sample-level visualisation of Zr–Hf–Te co-variation across the 121 reconstructed fruiting-body-level units (Figure 1). PC1 explained 93.4% of the total variance, PC2 explained 5.5% and PC3 explained 1.1%. PC1 and PC2 together accounted for 98.9% of the variance in the log10-transformed and standardised dataset. Because the PCA included only three strongly correlated variables, it primarily summarised the correlation structure and was not assigned independent source-related or mechanistic interpretation.

All three elements were strongly positively correlated with PC1, with variable–component correlations of 0.960 for Zr, 0.989 for Hf and 0.950 for Te. PC2 showed modest opposing contributions from Zr and Te, with correlations of −0.263 and 0.307, respectively, while the contribution of Hf to PC2 was limited (−0.039). PC3 explained little additional variance and was not interpreted substantively.

### 3.5. Contextual Topsoil Concentrations

Zr and Hf were detected in all 26 independent site-level topsoil composites (Table 3). The median Zr concentration was 11.0 mg kg^−1^ dw (IQR: 9.68–12.7 mg kg^−1^ dw), with a range of 6.65–17.7 mg kg^−1^ dw. The median Hf concentration was 1.37 mg kg^−1^ dw (IQR: 1.32–1.49 mg kg^−1^ dw), with a range of 1.18–1.75 mg kg^−1^ dw. Zr and Hf were strongly positively correlated across the 26 independent composites (Spearman’s *ρ* = 0.945, 95% BCa CI: 0.840–0.986, *p* < 0.001).

Te was below the working LoD in all 52 component analytical records and was therefore classified as <LoD in all 26 independent site-level composites. No quantitative descriptive statistics or correlation estimates were calculated for soil Te. The contrasting detection profile across mushrooms and contextual topsoils, particularly the high detection frequency of Te in mushrooms and the absence of measured Te concentrations exceeding the working LoD in topsoils, is shown in Supplementary Figure S2.

### 3.6. Exploratory Contextual Soil–Mushroom Comparisons

Exploratory same-element comparisons between mushroom and contextual topsoil concentrations were limited to the overlapping collection sites. Using site-level means, Spearman’s coefficients were negative and non-significant for Zr (*ρ* = −0.714, *p* = 0.111; *n* = 6 parks) and Hf (*ρ* = −0.200, *p* = 0.704; *n* = 6 parks). Median-based sensitivity comparisons were also negative and non-significant for Zr (*ρ* = −0.679, *p* = 0.094; *n* = 7 parks) and Hf (*ρ* = −0.250, *p* = 0.589; *n* = 7 parks).

These comparisons are inconclusive because of the small number of overlapping sites, the spatial mismatch between the contextual composite topsoils and mushroom collection points, and differences in the number and taxonomic composition of mushroom units among parks. Accordingly, they are not interpreted as evidence either for or against a direct relationship between contextual topsoil and mushroom concentrations.

Te was not evaluated quantitatively because it was below the working LoD in all contextual topsoil composites.

Apparent mushroom/contextual topsoil concentration ratios for Zr and Hf are reported in Supplementary Table S3 solely as descriptive area-level contextual indicators. Because the topsoil composites were not microsite- or rhizosphere-paired with the mushroom fruiting bodies, these ratios must not be interpreted as bioconcentration factors, transfer factors, bioaccumulation factors or mechanistic uptake coefficients.

## 4. Discussion

### 4.1. Occurrence of Zr, Hf and Te in Wild Mushrooms

The present study provides exploratory evidence of the occurrence of Zr, Hf and Te above the applicable working LoDs in acid digestates of cleaned wild mushroom fruiting bodies from Leicester/Leicestershire green spaces. Zr and Hf were detected in all reconstructed fruiting-body-level units, whereas Te was detected in 118/121 units. These results describe the analytical occurrence of the three elements in filtered mushroom digestates. They do not, by themselves, establish intracellular localisation, chemical speciation, environmental source or a specific biological uptake mechanism. These findings add to the small but growing literature showing that less commonly monitored elements can be quantified in fungal matrices when sufficiently sensitive multielement methods are used [4,8,9,10,11,14].

The contribution of this study is not that Zr, Hf or Te have never been reported in mushrooms, but rather that they remain poorly characterised relative to classical elements such as Cd, Pb, Hg, As, Cu and Zn. The present dataset provides a fruiting-body-level assessment of Zr, Hf and Te in a UK urban/peri-urban green space context, integrating taxon–site interpretation, explicit treatment of observations below the working LoD and contextual topsoil comparison.

This approach aligns with the broader use of mushrooms as bioindicators of environmental quality and biodiversity. The JRC reference mushroom framework emphasises that fungal element data can support bioindication when interpreted through robust taxonomy, adequate replication and ecological context [6]. The present study applies this principle to a focused panel of less regulated lithogenic and technology-associated elements.

### 4.2. Taxon–Site Patterns in Zr–Hf–Te Concentrations

A prominent descriptive finding was the variation in Zr–Hf–Te concentration profiles among the principal taxon–site groups. *Panaeolina foenisecii* collected at Abbey Park and Braunstone Park and *Mycena citrinomarginata* collected at Victoria Park showed the highest median concentrations across the three-element panel, whereas *Agaricus bitorquis* collected at St Augustine Road showed comparatively low and narrow concentration distributions. *Coprinopsis atramentaria* collected at Bradgate Park showed higher Zr and Hf concentrations than the *A. bitorquis* group, although the smaller number of reconstructed fruiting-body-level units requires cautious interpretation. Because most principal taxa were represented at a single collection site, these contrasts cannot be attributed to taxonomy independently of location, habitat or substrate conditions.

Species- and family-related differences in mushroom mineral composition are widely recognised [3,12]. Such variation may reflect fungal lifestyle, substrate use, mycelial distribution, element-specific biological handling and interactions with organic or mineral components of the substrate. However, the present sampling design does not permit these biological factors to be separated from site-related variation. The results should therefore be interpreted as concentration patterns in the sampled taxon–site combinations rather than as evidence of independent species-specific accumulation.

The comparatively high concentrations observed in *P. foenisecii* are of particular interest because this species is commonly associated with nutrient-rich grasslands, lawns, meadows, parks, gardens and roadsides [25]. Its ecological association with managed grassy habitats supports its relevance for further investigation in urban and peri-urban green spaces. Moreover, *P. foenisecii* was the only principal taxon collected at more than one site, and its site-level median concentrations were similar at Abbey Park and Braunstone Park. This descriptive consistency indicates that the observed profile was not restricted to a single park within the present dataset. Nevertheless, two collection sites are insufficient to establish a stable species-level trait or to classify *P. foenisecii* as a specific accumulator of Zr, Hf or Te. Confirmation would require targeted sampling across additional sites, seasons, habitats and substrates.

*Mycena citrinomarginata* also showed a marked Zr–Hf–Te profile within the 22 reconstructed fruiting-body-level units collected at Victoria Park. Because the exact microsubstrate of each fruiting body was not characterised and this taxon was represented at only one site, the relative contributions of fungal biology, substrate ecology, microhabitat conditions and fine organic or mineral material cannot be distinguished. The observed profile is therefore relevant within the sampled Victoria Park group but should not be generalised as a species-level characteristic.

By contrast, the *A. bitorquis* group collected at St Augustine Road showed lower and narrower concentration distributions. This finding demonstrates that elevated Zr–Hf–Te concentrations were not uniform across the sampled mushroom assemblages, although the contrast cannot be assigned exclusively to taxonomy. Overall, these results support a taxon–site-aware interpretation and identify specific combinations of taxa and locations for targeted multi-site investigation.

### 4.3. Zr–Hf Coherence in Mushrooms and Topsoils

The strong Zr–Hf association in both mushrooms and topsoils is geochemically plausible. Zr and Hf are closely related high-field-strength elements that commonly co-occur in resistant mineral phases, especially zircon and associated heavy minerals [15,16,17]. Their strong correlation in topsoil is therefore consistent with a common lithogenic control. Within the mushroom dataset, Hf concentrations frequently exceeded the corresponding Zr concentrations. At the overall level, the median Hf concentration was 270 ng g^−1^ dw, compared with 170 ng g^−1^ dw for Zr (Table 1), and the same relative pattern was observed across several of the principal taxon–site groups summarised in Table 2. Verification of analyte assignments, dilution factors and dry weight conversion calculations did not identify a systematic processing inconsistency that could explain this relationship. Nevertheless, because confirmatory measurements using alternative isotopes, matrix-matched reference materials or independent replicate digestions were not available, the observed Hf > Zr pattern should be interpreted cautiously. It is therefore reported as an analytical occurrence pattern requiring independent confirmation, rather than as evidence of selective fungal uptake, biological fractionation or a distinct environmental source.

The strong Zr–Hf correlation in mushrooms shows that the two elements co-varied in the analysed fruiting body digestates. However, it does not demonstrate a direct uptake pathway from topsoil to fruiting body. Zr and Hf may be present in biological tissues, associated with organic substrates, bound to fine mineral particles, retained on surfaces or incorporated through mechanisms that cannot be distinguished using bulk concentration data alone.

This distinction is important because Zr and Hf are often associated with mineral phases that are resistant to weathering and digestion. Their bioavailability may therefore be associated with minor reactive fractions rather than with total or pseudo-total soil concentrations. European geochemical evidence indicates that only a minor proportion of total Zr and Hf is extractable by aqua regia, consistent with the resistant nature of their host minerals [16]. Furthermore, Zr–Hf fractionation can occur in natural waters, colloids, authigenic phases and biologically influenced interfaces, showing that their behaviour in surface environments is more complex than simple lithogenic co-occurrence [17,18,19].

The exploratory site-level soil–mushroom comparisons did not show statistically significant associations for Zr or Hf. However, these analyses involved only six or seven overlapping parks and are inconclusive because of their limited statistical power, the spatial mismatch between contextual topsoil composites and mushroom collection points, and differences in the taxonomic composition of mushroom assemblages among parks. The results therefore cannot be used to determine whether mushroom concentrations were related to contextual topsoil conditions or to support any particular biological, substrate-related or microenvironmental mechanism.

### 4.4. Te Detected in Mushrooms but Below the Working LoD in Contextual Topsoils

Te was detected above the working LoD in nearly all reconstructed mushroom fruiting-body-level units but remained below the working LoD in all contextual topsoil composites. Because the topsoil composites were not microsite-paired with the mushroom fruiting bodies and no quantitative topsoil Te concentrations were available, Te was not included in soil–mushroom correlations or apparent concentration ratio calculations. This analytical matrix contrast should not be interpreted as evidence of Te uptake, enrichment, transfer or a particular environmental source.

This observation is consistent with the broader environmental chemistry of Te. Te is typically present at very low concentrations in natural waters, soils, sediments and airborne particles, and its determination often requires sensitive analytical methods [20]. Te occurs mainly as Te(IV) and Te(VI) in environmental systems, but its speciation in solid matrices remains poorly characterised [20,31]. The absence of measured Te concentrations exceeding the working LoD in contextual topsoils is consistent with its low environmental abundance and the analytical challenges associated with its determination in solid matrices.

Soil-to-crop studies demonstrate that Te can transfer from soils to plants, although transfer factors vary and are influenced by environmental conditions [22]. These data are useful as evidence that Te can enter biological matrices, but they cannot be directly extrapolated to wild mushrooms. Mushroom mycelia interact with substrates differently from plant roots, and saprotrophic fungi may access organic matter pools that are not represented by bulk topsoil measurements.

Several non-exclusive explanations may account for Te detection in mushrooms despite the absence of measured Te concentrations exceeding the working LoD in contextual topsoils. These include Te fractions below the working LoD of the contextual topsoil method, association with organic substrates, localised microhabitat heterogeneity, fine particulate inputs, atmospheric deposition, or fungal and microbial transformation or retention. Microbial transformation of Te has been reported in environmental microbiology, including formation of volatile organotellurium compounds by some bacteria and fungi [20]. However, such mechanisms were not tested in the present study and should be regarded as plausible background processes rather than demonstrated explanations.

Te was detected above the working LoD in nearly all mushroom digestates but remained below the working LoD in all contextual topsoil composites. The factors underlying this analytical contrast cannot be resolved from the available bulk concentration data.

### 4.5. Contextual Topsoil Interpretation

The topsoil data provide useful geochemical context but should not be overinterpreted as direct substrate controls. Zr and Hf were detected in all topsoils and were strongly correlated, consistent with lithogenic co-occurrence. Te remained below the working LoD in every contextual topsoil composite, consistent with its low environmental abundance and analytical complexity. However, the topsoil samples were composite-derived area-level materials, whereas mushroom fruiting bodies may reflect microscale substrate conditions, organic matter, mycelial depth and taxon-specific biology.

For national-scale contextual comparison, the median-to-Atlas ratios were 0.045 for Zr and 0.197 for Hf, based on the corresponding reference values reported in the *Advanced Soil Geochemical Atlas of England and Wales* [15]. However, the Atlas values were generated using total X-ray fluorescence, whereas the present topsoil results represent acid-recoverable concentrations obtained using reverse aqua regia digestion. These values are therefore presented solely as descriptive geochemical context and should not be interpreted as evidence of enrichment or depletion.

This scale mismatch is central to the interpretation. Falandysz and Borovička [3] emphasised that macrofungal accumulation depends on multiple natural, taxonomic and environmental factors, and that the relationship between soil concentration and fruiting body concentration is often complex. Campos and Tejera [2] similarly showed that fungal nutritive behaviour and mineral substrate properties influence element occurrence in sporocarps. Zocher et al. [5] further demonstrated that fungal tissues and associated substrates may show related but not identical element patterns, with intra-fruiting body distribution also contributing to variability. Ivanić et al. [23] also showed that soil, mosses and mushrooms may share broad multielement patterns while retaining matrix-specific signals.

For these reasons, the apparent mushroom/contextual topsoil concentration ratios calculated for Zr and Hf are retained solely as descriptive area-level contextual indicators in Supplementary Table S3. They must not be interpreted as bioconcentration factors, transfer factors, bioaccumulation factors or mechanistic uptake coefficients. This distinction is particularly important because the contextual topsoil composites were not microsite- or rhizosphere-paired with the mushroom fruiting bodies and may not represent the organic or mineral substrates accessed by the underlying mycelia.

### 4.6. Relevance of the Urban and Peri-Urban Green Space Setting

The urban/peri-urban setting provides ecological context but does not by itself indicate an anthropogenic source. Public green spaces may receive inputs from soil disturbance, dust, vegetation management, organic debris, traffic-adjacent particles and localised historical land use. However, Zr and Hf have strong lithogenic controls, and Te remained below the working LoD in all contextual topsoil composites. Therefore, the present data do not support attribution to any single source.

Previous biomonitoring work along major roads has shown that fungi, soils and lichens may respond differently to environmental inputs and that fungal metal concentrations can vary substantially among species and sites [32]. Multielement mushroom studies near traffic-affected environments also show that fungal matrices can contain a broad range of elements, including less frequently measured endpoints such as Te, but that species and substrate effects remain important [4]. This supports the broader interpretation that fungi are useful biological matrices but require taxon-aware and matrix-aware interpretation. In the present study, the most defensible statement is that Zr–Hf–Te signatures were observed in mushrooms from urban and peri-urban green spaces, not that these signatures necessarily reflect urban contamination.

### 4.7. Strengths and Limitations

The principal strengths of this study are its focus on under-characterised elements, reconstruction of mushroom data at fruiting body level, explicit treatment of observations below the working LoD, taxon–site interpretation and integration of contextual soil information without assuming direct uptake. Analytical quality control supported instrumental calibration consistency and repeatability for Zr, Hf and Te in mushrooms and for Zr and Hf in topsoils.

The reconstruction reduced cap–stipe pseudoreplication but did not yield mass-weighted whole-fruiting-body concentrations. Paired cap–stipe analyses involving 32 fruiting bodies did not identify significant differences for Zr (BH-adjusted *q* = 0.664; rank biserial correlation = 0.159, 95% BCa CI: −0.269 to 0.523) or Hf (*q* = 0.775; rank biserial correlation = 0.061, 95% BCa CI: −0.348 to 0.450). Te concentrations were moderately higher in caps than in stipes, with median concentrations of 46.3 and 42.5 ng g^−1^ dw, respectively, a median within-pair difference of 4.29 ng g^−1^ dw, *q* = 0.017 and a rank biserial correlation of 0.549 (95% BCa CI: 0.129 to 0.822). All Te measurements included in the paired analysis were above the working LoD. The Te result was considered exploratory because 22 of the 32 pairs (68.8%) corresponded to *Agaricus bitorquis* collected at St Augustine Road (Supplementary Table S1).

The study also has limitations. The sampling was opportunistic and taxon representation was uneven. Taxon and collection site were closely associated for most principal groups, preventing estimation of independent site-adjusted taxonomic effects. Some taxa were represented by small numbers of fruiting bodies and should be interpreted descriptively only. Rural representation was limited and should not be used as a broad rural comparator. Topsoil samples were contextual composites rather than microsite-paired substrates. All Te measurements in the contextual topsoils were below the working LoD, preventing quantitative soil Te summaries or soil–mushroom Te comparisons. Finally, the study did not determine Te speciation, Zr/Hf mineral association, particle adherence, in vitro bioaccessibility or subcellular localisation. These analyses would be valuable in future work.

## 5. Conclusions

This study provides an exploratory occurrence profile of acid-recoverable Zr, Hf and Te in wild mushroom fruiting bodies collected from urban and peri-urban green spaces in Leicester and Leicestershire. Zr and Hf were detected above their respective working LoDs in all 121 fruiting-body-level units, while Te was detected in 118 units. The strong associations observed among park-level concentrations, together with the multivariate analysis, revealed a consistent pattern of co-variation across the three elements. This coherence describes a consistent multielement pattern in the sampled mushrooms, although it does not, by itself, establish a common environmental source, uptake pathway or biological process.

Concentration profiles differed among the principal taxon–site groups. Comparatively elevated concentrations were observed in *Panaeolina foenisecii* from Abbey and Braunstone Parks and in *Mycena citrinomarginata* from Victoria Park, whereas *Agaricus bitorquis* from St. Augustine Road showed lower and less variable concentrations. Because most represented taxa were associated with a single collection site, these findings are best interpreted as taxon–site patterns rather than as independent species-specific effects. They nevertheless show that both biological and local environmental context should be considered when wild mushrooms are used to investigate less-characterised elements.

The contextual topsoil data showed a strong relationship between Zr and Hf, whereas Te remained below the working LoD in every topsoil composite. The available soil–mushroom comparisons did not identify a consistent association for Zr or Hf and cannot resolve soil-to-mushroom transfer because the topsoil composites were not paired with the immediate substrates explored by the fungal mycelia. The contrasting occurrence of Te in mushroom and topsoil digestates therefore remains an important unresolved analytical and environmental observation rather than evidence of enrichment or uptake.

Overall, the results establish a valuable baseline for Zr, Hf and Te in wild mushrooms and support the inclusion of macrofungi in the investigation of under-characterised lithogenic and technology-associated elements. Future studies should combine replicated taxa across multiple locations with microsite-paired substrates, matrix-matched analytical validation and targeted characterisation of mineral particles and Te speciation. Such an approach will help determine how fungal identity, substrate accessibility, local geochemistry and particle-associated inputs contribute to the multielement patterns identified here.

## Figures and Tables

**Table 1:** Detection frequency and descriptive statistics for Zr, Hf and Te in reconstructed mushroom fruiting-body-level units.

Element	*N*	Detected *n* (%)	<LoD (%)	LoD (ng g^−1^ dw)	Median (IQR) (ng g^−1^ dw)	Range (ng g^−1^ dw)	Detected Range (ng g^−1^ dw)	Mean	GM	P95	P97.5
Zr	121	121/121 (100%)	0%	0.294	170 (61–380)	28–3200	28–3200	320	170	1100	1500
Hf	121	121/121 (100%)	0%	0.823	270 (99–850)	53–6000	53–6000	680	330	2800	3700
Te	121	118/121 (97.5%)	2.5%	5.269	110 (46–420)	<5.269–3300	6.8–3300	350	140	1700	2100

Concentrations are expressed as ng g^−1^ dry weight. Three Te observations in mushrooms were below the working LoD (3/121; 2.5%). The lower bound of the Te working range is shown as <5.269 ng g^−1^ dw because three observations were below the working LoD; the detected range is reported separately. The Te arithmetic and geometric means were calculated using constrained ROS estimates for the three observations below the working LoD; the median, interquartile range and upper percentiles were invariant to their exact values. Medians, interquartile ranges and percentile-based summaries are emphasised because the distributions were right-skewed.

**Table 2:** Descriptive concentrations of Zr, Hf and Te in the principal mushroom taxon–site groups represented by at least five reconstructed fruiting-body-level units.

Taxon	*N*	Zr Median (IQR)	Zr Range	Zr Detected	Hf Median (IQR)	Hf Range	Hf Detected	Te Median (IQR)	Te Range	Te Detected
*Panaeolina foenisecii*	28	390 (220–660)	32–3200	28/28	950 (530–1600)	97–4900	28/28	540 (270–930)	22–2300	28/28
*Mycena citrinomarginata*	22	390 (300–500)	120–2000	22/22	730 (630–870)	240–6000	22/22	360 (290–470)	<5.269–3300	21/22
*Coprinopsis atramentaria*	8	260 (79–450)	44–850	8/8	520 (210–1100)	96–2000	8/8	100 (77–560)	42–1100	8/8
*Agaricus bitorquis*	22	56 (50–62)	38–140	22/22	98 (98–99)	97–110	22/22	44 (41–46)	18–49	22/22
Unknown/unidentified taxon	17	89 (72–140)	60–300	17/17	110 (100–160)	99–490	17/17	54 (51–59)	6.8–280	17/17

Values are expressed as ng g^−1^ dry weight and are reported to two significant figures, except that the working LoD was retained where a concentration range included a value below the LoD. The results describe the sampled taxon–site groups and should not be interpreted as independent taxonomic effects because most principal taxa were represented at a single collection site. The complete distribution of taxa across collection sites is provided in Supplementary Table S4. The unknown/unidentified group is retained for descriptive completeness but is not interpreted taxonomically. Te was detected in all listed groups except *Mycena citrinomarginata*, for which 21/22 units were detected; its Te range is therefore reported with a <LoD lower bound.

**Table 3:** Detection frequency and descriptive statistics for Zr, Hf and Te in contextual topsoils.

Element	*N*	Detected *n* (%)	<LoD (%)	LoD (mg kg^−1^ dw)	Median (IQR) (mg kg^−1^ dw)	Range (mg kg^−1^ dw)	Mean	GM	P95	P97.5
Zr	26	26/26 (100%)	0	0.0131	11.0 (9.68–12.7)	6.65–17.7	11.2	11.0	14.3	15.7
Hf	26	26/26 (100%)	0	0.0278	1.37 (1.32–1.49)	1.18–1.75	1.40	1.39	1.55	1.63
Te	26	0/26 (0%)	100	0.0556	Not estimated	All < LoD	/	/	/	/

Soil concentrations are expressed as mg kg^−1^ dry weight. Environmental summaries are based on *N* = 26 independent site-level composites. For Zr and Hf, each composite value was calculated as the arithmetic mean of two composite-derived analytical/subsampling records; the 52 component records were retained only for analytical traceability and QA/QC. Te was below the working LoD in all 52 component records and was therefore classified as <LoD in all 26 composites; no quantitative descriptive statistics were estimated for Te. / = not estimated.

## Data Availability

The data presented in this study are available in the Open Science Framework (OSF) repository, *Leicester topsoil and wild mushroom metal dataset*, at https://osf.io/xbrd7/overview?view_only=750aba9f380044e38ce5c9162e0d4020 (accessed on 1 July 2026). The repository includes curated CSV files containing raw and final calculated concentrations, limits of detection (LoDs) and below-LoD flags for the topsoil and wild mushroom datasets. The dataset is released under the Creative Commons Attribution 4.0 International licence (CC BY 4.0).

## Supplementary Materials

The following supporting information can be downloaded at https://www.mdpi.com/article/10.3390/jox16040139/s1, Figure S1: Study framework and spatial distribution of wild mushroom collection sites and contextual topsoil sampling sites in Leicester and Leicestershire; Figure S2: Detection frequencies for Zr, Hf and Te in reconstructed wild mushroom fruiting-body-level units and contextual topsoil composites; Table S1: Sensitivity analyses for fruiting body reconstruction and paired cap–stipe comparisons; Table S2: Analytical calibration, detection limits and quality control performance for Zr, Hf and Te in mushroom and topsoil digestates; Table S3: Apparent area-level mushroom/contextual topsoil concentration ratios for Zr and Hf; Table S4: Distribution of reconstructed fruiting-body-level units by taxon and collection site (*N* = 121); Table S5: Site-level mushroom concentration medians and exploratory inter-element correlations.
