# Supplementary material for: Zirconium, Hafnium and Tellurium Signatures in Wild Mushrooms from Leicester and Leicestershire, England
Source: J Xenobiot. 2026 Jul 29;16(4):139. doi: 10.3390/jox16040139 (PMC13514944; doi:10.3390/jox16040139)
Supplement: Supplementary file 1 [file jox-16-00139-s001.zip › jox-4422890-supplementary.pdf]

Supplementary Materials: Zirconium, Hafnium and Tellurium Signatures in Wild Mushrooms from Leicester and Leicestershire, England

Antonio Peña-Fernández, Tomás Cámara-Pastor, M. Ángeles Peña Fernández and M. Carmen Lobo-Bedmar

Figure S1. Study framework and spatial distribution of wild mushroom collection sites and contextual topsoil sampling sites in Leicester and Leicestershire. Mushroom data were analysed as reconstructed fruiting-body-level units (N = 121). Topsoil data represent 26 independent site-level composites generated from 850 field increments; the 52 composite-derived analytical/subsampling records were retained only for analytical traceability and QA/QC. Accordingly, soil–mushroom comparisons are interpreted as contextual site-level associations rather than as microsite- or rhizosphere-paired assessments of soil-to-mushroom transfer.

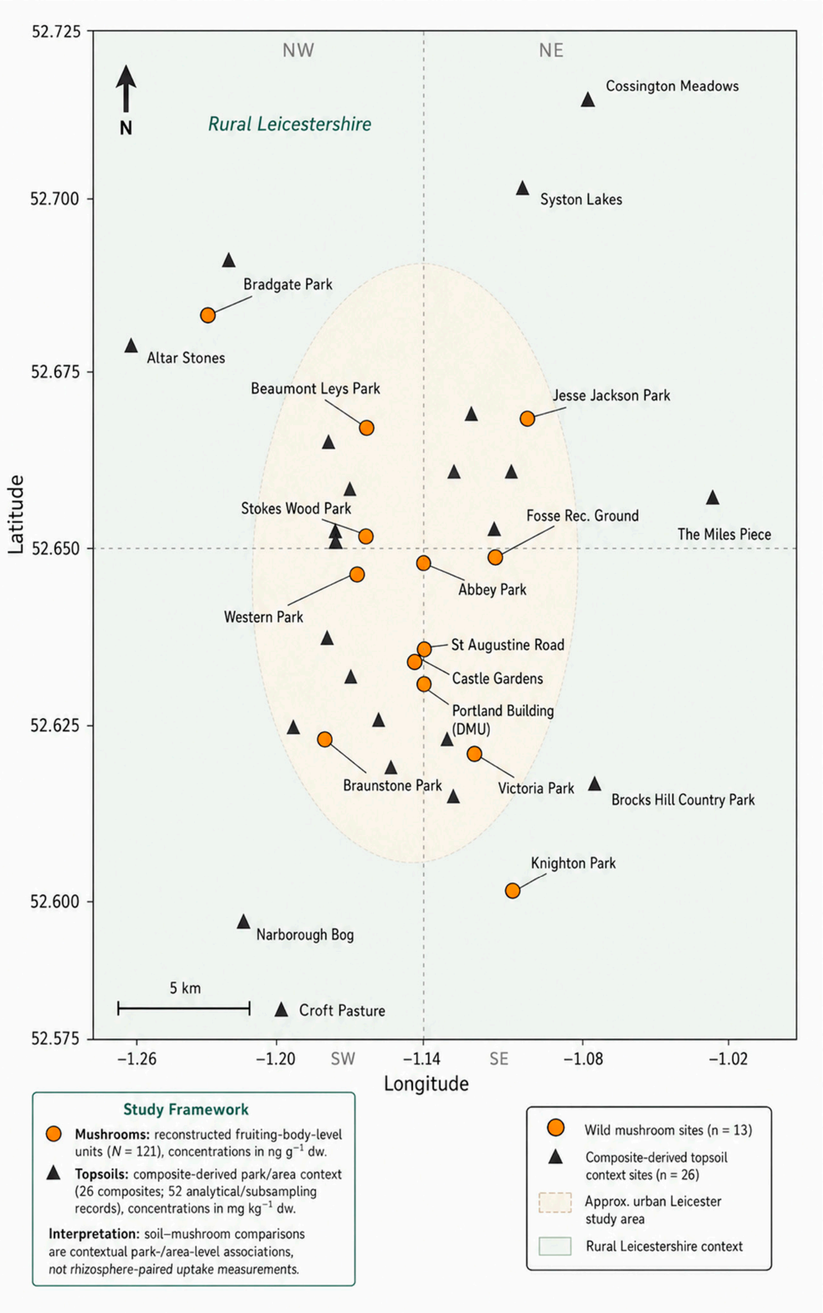

Figure S2. Detection frequencies for Zr, Hf and Te in reconstructed wild mushroom fruiting-body-level units and contextual topsoil composites. Mushroom data are based on 121 reconstructed fruiting-body-level units. Topsoil data are based on 26 independent site-level composites; two analytical/subsampling records were available for each composite and were averaged for Zr and Hf. Zr and Hf were detected above their respective working LoDs in all mushroom units and all 26 topsoil composites. Te was detected in 118/121 mushroom units but remained below the working LoD in all 52 component topsoil records and, consequently, in all 26 site-level composites.

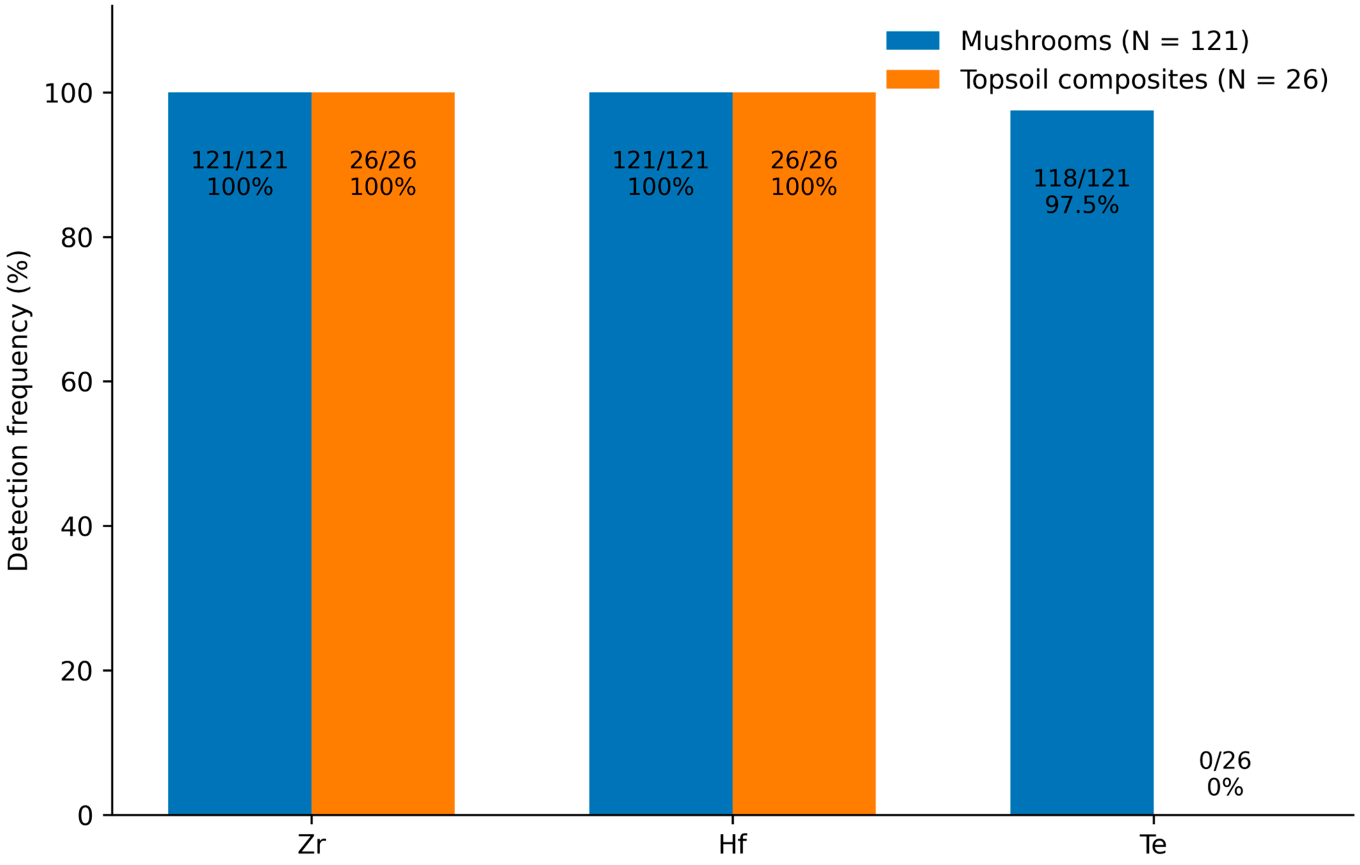

Supplementary Table S1. Sensitivity analyses for fruiting-body reconstruction and paired cap–stipe comparisons.

Panel A. Descriptive sensitivity by analytical representation.

Analytical representation	Element	n	Median	Q1	Q3	IQR	Detected minimum	Maximum	<LoD (%)
Whole fruiting bodies analysed directly	Zr	89	284.0	135.0	463.0	328.0	32.4	3240.0	0.0
	Hf	89	598.0	155.0	1000.0	846.0	96.1	6010.0	0.0
	Te	89	280.0	74.4	544.0	470.0	6.76	3350.0	3.4
Unweighted arithmetic means of paired cap–stipe records	Zr	32	53.2	43.8	60.6	16.8	28.3	136.0	0.0
	Hf	32	98.0	97.1	99.1	2.03	52.9	269.0	0.0
	Te	32	44.6	40.9	47.2	6.35	18.4	155.0	0.0
Caps	Zr	32	48.3	42.5	64.7	22.2	8.17	166.0	0.0
	Hf	32	98.0	96.9	98.7	1.83	9.67	374.0	0.0
	Te	32	46.3	44.1	50.4	6.27	5.4	218.0	0.0
Stipes	Zr	32	48.6	40.7	59.3	18.7	30.2	125.0	0.0
	Hf	32	97.9	97.2	99.0	1.82	95.8	181.0	0.0
	Te	32	42.5	37.8	48.1	10.3	12.5	98.6	0.0

Panel B. Paired Wilcoxon signed-rank analyses for the 32 matched cap–stipe pairs.

Element	n pairs	Cap median	Stipe median	Median cap–stipe difference	Wilcoxon statistic	*p* value	BH-adjusted *q*	Rank-biserial correlation	95% BCa CI
Zr	32	48.3	48.6	4.21	222	0.443	0.664	0.159	−0.269–0.523

Hf	32	98.0	97.9	0.0975	248	0.775	0.775	0.061	−0.348–0.450
Te	32	46.3	42.5	4.29	119	0.006	0.017	0.549	0.129–0.822

Concentration statistics in Panel A, together with the cap and stipe medians and median within-pair differences in Panel B, are expressed as ng g^{-1} dw. The median cap–stipe difference represents the median of the individual within-pair differences and was not calculated by subtracting the two marginal medians. Panel A is descriptive because the analytical-representation groups differ substantially in their taxonomic and site composition. General and taxon-level Te summaries that included observations below the working LoD were estimated using NADA/ROS. In Panel B, all Te measurements from the 32 matched cap–stipe pairs were above the working LoD; therefore, no observation below the working LoD was included in the Wilcoxon signed-rank test. The Te result should nevertheless be considered exploratory because 22 of the 32 pairs (68.8%) corresponded to *Agaricus bitorquis* collected at St Augustine Road. Bias-corrected and accelerated 95% confidence intervals for the rank-biserial correlations were calculated using 10,000 bootstrap resamples, with the matched fruiting body as the resampling unit.

Supplementary Table S2. Analytical calibration, detection limits and quality-control performance for Zr, Hf and Te in mushroom and topsoil digestates.

Matrix	Element	Calibration range	Instrumental DL	Working LoD	QC solution	QC measured mean ± SD	Recovery (%)	RSD (%)
Mushroom digestates	Zr	Ten-point calibration; upper standard 77.759 μg L^{-1}	0.007 μg L^{-1}	0.294 ng g^{-1} dw	QCB 280	286.97 ± 9.39 μg L^{-1}	102.49	3.27
	Hf		0.001 μg L^{-1}	0.823 ng g^{-1} dw	QCB 280	285.53 ± 17.46 μg L^{-1}	101.97	6.11
	Te		0.014 μg L^{-1}	5.269 ng g^{-1} dw	QCB 280	302.13 ± 20.34 μg L^{-1}	107.90	6.73
Topsoil digestates	Zr	Ten-point calibration; nominal upper standard 117 μg L^{-1}	0.005 μg L^{-1}	0.0131 mg kg^{-1} dw	QCB 274	263.99 ± 4.10 μg L^{-1}	96.35	1.55
	Hf		0.002 μg L^{-1}	0.0278 mg kg^{-1} dw	QCB 274	270.13 ± 1.50 μg L^{-1}	98.59	0.56
	Te		0.014 μg L^{-1}	0.0556 mg kg^{-1} dw	QCB 274	275.85 ± 15.65 μg L^{-1}	100.67	5.68

DL, instrumental detection limit; LoD, working limit of detection; dw, dry weight; RSD, relative standard deviation. Calibration ranges and instrumental DLs are expressed for the analysed digestate solutions. Working dry-weight LoDs were calculated by converting the instrumental DLs according to the recorded sample mass, final digest volume and dilution factor. The independent aqueous QC solutions were automatically diluted twofold before analysis; their introduced concentrations remained above the corresponding upper calibration standards, and the reported QC values therefore represent approximate instrumental estimates obtained by extrapolation of the linear calibration models. These aqueous QC results assess instrumental performance and repeatability but do not independently evaluate digestion efficiency. Four initial 2% v/v HNO_3 wash solutions in the mushroom analytical sequence gave mean concentrations of 0.03751 ± 0.00018 μg L^{-1} for Zr, 0.17431 ± 0.00049 μg L^{-1} for Hf and 0.10208 ± 0.00315 μg L^{-1} for Te. No blank subtraction was applied. The instrumental DLs shown in the table are those reported in the archived Syngistix calibration outputs and were used to calculate the working dry-weight LoDs.

Supplementary Table S3. Apparent area-level mushroom/contextual-topsoil concentration ratios for Zr and Hf.

Element	Park	Contextual topsoil composite concentration (mg kg^{-1} dw)	Mushroom median concentration (ng g^{-1} dw)	Apparent mushroom/topsoil concentration ratio
Zr	Abbey Park	6.65	403	0.0606
	Bradgate Park	8.52	265	0.0310
	Braunstone Park	10.2	357	0.0349
	Knighton Park	12.7	57.3	0.00450
	Stokeswood Park	9.67	235	0.0244
	Victoria Park	9.22	388	0.0421
	Western Park	9.31	60.5	0.00650
Hf	Abbey Park	1.18	966	0.819
	Bradgate Park	1.25	520	0.417
	Braunstone Park	1.36	926	0.681
	Knighton Park	1.46	117	0.0805
	Stokeswood Park	1.32	370	0.281
	Victoria Park	1.33	731	0.551
	Western Park	1.25	114	0.0909

Ratios were calculated after unit harmonisation using the concentration of the independent contextual topsoil composite available for each park and the corresponding median mushroom concentration. Each topsoil composite concentration represents the arithmetic mean of its two composite-derived analytical/subsampling records. These ratios are retained solely as descriptive area-level contextual indicators. Because the topsoil composites were not microsite- or rhizosphere-paired with the mushroom fruiting bodies, the ratios must not be interpreted as bioconcentration factors, transfer factors, bioaccumulation factors or mechanistic uptake coefficients. Te was excluded because it was below the working LoD in all contextual topsoil records and composites.

Supplementary Table S4. Distribution of reconstructed fruiting-body-level units by taxon and collection site (N = 121).

Taxon	AP	BLP	BrP	BnP	CG	FRG	JJP	KP	DMU	SAR	SWP	VP	WP	Total
Agaricus arvensis	0	0	0	0	0	1	0	0	0	0	0	0	1	2
Agaricus bitorquis	0	0	0	0	0	0	0	0	0	22	0	0	0	22
Agaricus nivescens	0	0	0	0	0	0	1	0	0	0	0	0	0	1
Armillaria mellea	0	0	0	0	0	0	0	3	0	0	0	0	0	3
Coprinopsis atramentaria	0	0	8	0	0	0	0	0	0	0	0	0	0	8
Hypholoma fasciculare	0	0	0	0	0	0	0	1	0	0	0	0	0	1
Leucoagaricus leucothites	0	0	0	0	0	0	0	0	1	0	0	0	0	1
Marasmius oreades	0	0	0	0	0	0	4	0	0	0	0	0	0	4
Mycena citrinomarginata	0	0	0	0	0	0	0	0	0	0	0	22	0	22
Panaeolina foenisecii	23	0	0	5	0	0	0	0	0	0	0	0	0	28
Pholiota adiposa	0	0	0	0	0	0	0	0	0	0	2	0	0	2
Pholiota gummosa	0	0	0	0	0	0	0	0	0	0	1	0	0	1
Psathyrella corrugis	0	1	0	0	0	0	0	0	0	0	0	0	0	1
Psathyrella fatua	0	0	0	0	0	0	0	0	0	0	3	0	0	3
Psathyrella sublatispora	0	0	0	0	0	0	0	0	0	0	1	0	0	1
Unknown/unidentified taxon	0	0	0	0	17	0	0	0	0	0	0	0	0	17
Vascellum pratense	0	0	0	0	0	0	2	0	0	0	0	0	0	2
Xerocomus porosporus	0	0	0	0	0	0	0	0	0	0	0	0	2	2

Site abbreviations: AP, Abbey Park; BLP, Beaumont Leys Park; BrP, Bradgate Park; BnP, Braunstone Park; CG, Castle Garden; FRG, Fosse Recreation Ground; JJP, Jesse Jackson Park; KP, Knighton Park; DMU, Portland Building, De Montfort University; SAR, St Augustine Road; SWP, Stokeswood Park; VP, Victoria Park; WP, Western Park. Counts refer to reconstructed fruiting-body-level analytical units. Among the principal groups with $n \geq 5$, only *Panaeolina foenisecii* occurred at more than one site. Consequently, concentration differences among the principal groups are interpreted descriptively as taxon–site patterns rather than as independent taxonomic effects.

Supplementary Table S5. Site-level mushroom concentration medians and exploratory inter-element correlations.

Panel A. Park-level medians.

Collection site	*n* units	*n* taxa	Zr median (ng g^{-1} dw)	Hf median (ng g^{-1} dw)	Te detected *n* (%)	Te median (ng g^{-1} dw)
Abbey Park	23	1	403	966	23/23 (100.0%)	539
Beaumont Leys Park	1	1	287	567	1/1 (100.0%)	186
Bradgate Park	8	1	265	520	8/8 (100.0%)	103
Braunstone Park	5	1	357	926	5/5 (100.0%)	535
Castle Garden	17	1	89.2	107	17/17 (100.0%)	54.5
Fosse Rec. Ground	1	1	37.3	97.1	1/1 (100.0%)	52.7
Jesse Jackson Park	7	3	53.5	110	6/7 (85.7%)	52.2
Knighton Park	4	2	57.3	117	4/4 (100.0%)	51.6
Portland Building, DMU	1	1	89.5	97.6	1/1 (100.0%)	37
St Augustine Road	22	1	56.1	98	22/22 (100.0%)	44.3
Stokeswood Park	7	4	235	370	6/7 (85.7%)	173
Victoria Park	22	1	388	731	21/22 (95.5%)	356
Western Park	3	2	60.5	114	3/3 (100.0%)	59.3

Panel B. Spearman correlations among park-level medians (N = 13 collection sites).

Comparison	*N* parks	Spearman's ρ	95% BCa CI	*p* value	BH-adjusted *q*
Zr-Hf	13	0.863	0.381–0.983	<0.001	<0.001

Zr-Te	13	0.819	0.269–0.961	<0.001	<0.001
Hf-Te	13	0.885	0.419–0.994	<0.001	<0.001

The three Te observations below the working LoD occurred below the detected concentrations defining the medians of their respective parks; therefore, the Te medians and their rank order were invariant to the unknown values below the working LoD. Correlations are exploratory because parks are aggregate ecological units with unequal numbers and taxonomic compositions of fruiting bodies. Bias-corrected and accelerated 95% confidence intervals (CI) were calculated using 10,000 bootstrap resamples, with the collection site as the resampling unit.
